# Arsenic stress triggers active exudation of arsenic–phytochelatin complexes from *Lupinus albus* roots

**DOI:** 10.1093/jxb/erae272

**Published:** 2024-06-12

**Authors:** Adrien Frémont, Eszter Sas, Mathieu Sarrazin, Jacques Brisson, Frédéric Emmanuel Pitre, Nicholas James Beresford Brereton

**Affiliations:** Institut de recherche en biologie végétale, Département de sciences biologiques, Université de Montréal, 4101 Sherbrooke Est, Montréal, QC H1X 2B2, Canada; Environmental Genomics and Systems Biology, Lawrence Berkeley National Laboratory, Berkeley, CA 94720, USA; Institut de recherche en biologie végétale, Département de sciences biologiques, Université de Montréal, 4101 Sherbrooke Est, Montréal, QC H1X 2B2, Canada; Collège de Maisonneuve CÉPROCQ, 6220 Sherbrooke Est, Montréal, QC H1N 1C1, Canada; Institut de recherche en biologie végétale, Département de sciences biologiques, Université de Montréal, 4101 Sherbrooke Est, Montréal, QC H1X 2B2, Canada; Institut de recherche en biologie végétale, Département de sciences biologiques, Université de Montréal, 4101 Sherbrooke Est, Montréal, QC H1X 2B2, Canada; Montreal Botanical Garden, 4101 Sherbrooke Est, Montreal, QC H1X 2B2, Canada; School of Biology and Environmental Science, University College Dublin, Belfield, Dublin 4, Ireland; University of Porto, Portugal

**Keywords:** Arsenic, arsenic–phytochelatin complexes, exclusion, *Lupinus albus*, phytochelatin, phytoremediation, rhizosphere, root exudates, sequestration, soil pollution

## Abstract

Arsenic (As) contamination of soils threatens the health of millions globally through accumulation in crops. While plants detoxify As via phytochelatin (PC) complexation and efflux of arsenite from roots, arsenite efflux mechanisms are not fully understood. Here, white lupin (*Lupinus albus*) was grown in semi-hydroponics, and exudation of glutathione (GSH) derivatives and PCs in response to As was measured using LC-MS/MS. Inhibiting synthesis of the PC precursor GSH with l-buthionine sulfoximine (BSO) or ABC transporters with vanadate drastically reduced (>22%) GSH derivative and PC_2_ exudation, but not PC_3_ exudation. This was accompanied by As hypersensitivity in plants treated with BSO and moderate sensitivity with vanadate treatment. Investigating As–PC complexation revealed two distinct As–PC complexes, As bound to GSH and PC_2_ (GS–As–PC_2_) and As bound to PC_3_ (As–PC_3_), in exudates of As-treated lupin plants. Vanadate inhibited As–PC exudation, while BSO inhibited both the synthesis and exudation of As–PC complexes. These results demonstrate a role for GSH derivatives and PC exudation in lupin As tolerance and reveal As–PC exudation as a new potential mechanism contributing to active As efflux in plants. Overall, this study uncovers insights into rhizosphere As detoxification with potential to help mitigate pollution and reduce As accumulation in crops.

## Teaser

Active root exudation of arsenic–phytochelatin complexes in white lupin provides evidence of a new mechanism of arsenic efflux in plants, indicating novel pathways for phytoremediation and food safety.

## Introduction

Arsenic (As) is a major soil contaminant affecting environment and human health. Contamination of soil and groundwater is widespread, with elevated As concentrations resulting from natural weathering and biological activity, as well as anthropogenic activities such as mining and agriculture (Patel *et al.*, 2023). With an estimated 2.8 million contaminated sites in Europe and 20 Mha of farmland affected by heavy metals in China, extensive soil pollution is putting substantial pressure on agricultural soils (FAO and ITPS, 2015). Because plants readily take up mobile As from the soil and translocate it to above-ground tissues, dietary intake presents a major exposure route to humans, potentially threatening millions with a range of adverse health effects from As poisoning. In particular, accumulation in major food crops such as rice is an international concern (Zhao *et al.*, 2010a). Therefore, understanding the specific processes governing As bioavailability, uptake, and detoxification in plants is critical for developing mitigation strategies. One crucial plant–soil interaction thought to help plants adapt to and influence As-challenged soil is the release of diverse metabolites from roots (root exudates) which can markedly impact soil chemistry and microbiota to influence contaminant tolerance (Podar and Maathuis, 2022). However, the molecular mechanisms underlying root exudate-mediated As detoxification are not fully elucidated.

In aerobic soils, plants predominantly encounter As in the form of arsenate [As(V)] which enters roots through phosphate transporters (Asher and Reay, 1979; Ullrich-Eberius *et al.*, 1989), while arsenite [As(III)] is most prevalent in anaerobic soils and enters roots through aquaporin channels (Bienert *et al.*, 2008).

Arsenic detoxification in plants involves the reduction of As(V) to As(III) by arsenate reductases such as ACR2 and HAC1, identified in both hyperaccumulating and non-hyperaccumulating plant species (Li *et al.*, 2016). The subsequent tolerance mechanisms have been largely studied in the As hyperaccumulator *Pteris vittata* (Ma *et al.*, 2001; Lombi *et al.*, 2002; Su *et al.*, 2008). In *P. vittata*, the reduced As(III) is rapidly transported to above-ground tissues, where free As(III) is sequestered in vacuoles (Zhao *et al.*, 2009). In contrast, non-hyperaccumulating species preferentially eliminate up to 90% of internally reduced As(III) back into the external medium, partly through aquaporin channels but also through as yet unidentified pathways (Zhao *et al.*, 2010b), while the remaining intracellular As(III) is complexed with phytochelatins (PCs) (Schmöger *et al.*, 2000).

PCs are oligomers derived from glutathione (GSH) and comprised of (γ-glutamylcysteinyl)_*n*_ glycine units, typically with *n* between 2 and 6. PCs can form stable complexes with As through coordination with cysteine thiol groups, including As–PC_3_, As–(PC_2_)_2_, and GS–As–PC_2_ (Raab *et al.*, 2004). Other toxic metals such as cadmium can also complex with PCs and GSH in a similar manner (Lux *et al.*, 2011). The formation of these complexes plays a critical role in metal(loid) detoxification and stabilization in plants. Liu *et al.* (2010) found up to 70% of intracellular As(III) bound to PCs in *Arabidopsis thaliana* roots. Mutants deficient in PC synthesis, such as *cad1-3*, exhibit 10- to 20-fold greater sensitivity to arsenate, measured by seedling biomass production, compared with wild-type plants (Ha *et al.*, 1999). Furthermore, inhibition of the PC precursor GSH using l-buthionine sulfoximine (BSO) induces As hypersensitivity across diverse plant species (Meharg and Hartley-Whitaker, 2002).

The ultimate phase of As detoxification in non-hyperaccumulating plants is thought to occur through ATP-dependent vacuolar loading of As–PC complexes, mediated by ABC transporters localized to the tonoplast, as evidenced by inhibition of As–PC vacuolar sequestration with the ABC transport inhibitor vanadate (Song *et al.*, 2010). However, recent evidence also points to extracellular roles for PCs in As detoxification. PC_2_ was found in root exudates of *Lupinus albus*, suggesting As complexation in the rhizosphere or PC complex-mediated efflux of As(III) directly from roots (Frémont *et al.*, 2022). Elucidating the mechanisms and roles of PC exudation is important for harnessing the potential of root exudates to mitigate As environmental and health damage, yet a number of challenging methodological hurdles need to be overcome.

Current methods for capturing root exudates largely utilize hydroponic and aeroponic cultivation systems. While these substrate-free growth methods provide easy access to roots and root exudates, they lack the physical properties of soils that influence root architecture and physiology (Oburger and Jones, 2018). Soil-like substrates such as silica sand may offer advantages, providing mechanical support and resistance to root growth that feed back to modulate root morphology and exudate composition, while still maintaining inert properties to limit interference from soil organic particles (Sasse *et al.*, 2020). Beyond experimental limitations, chemical characterization of root exudates also faces several hurdles, particularly due to the intricate chemical interactions occurring between As and root exudates, which may produce transient compounds critical for As detoxification (Bluemlein *et al.*, 2009). Analytical approaches such as LC-MS/MS have enabled detection of previously unknown metal–organic complexes involved in metal detoxification in the rhizosphere, such as zinc–nicotianamine (Tsednee *et al.*, 2014). Interestingly, similar mechanisms of extracellular metal detoxification have been identified in microbes, which involve the intracellular production and efflux of the As complex 1-arseno-3-phosphoglycerate, providing a novel As detoxification pathway (Chen *et al.*, 2016). Therefore, coupling physiologically relevant growth systems with selective, high-resolution analytical techniques may shed light on specialized plant exudation mechanisms for As detoxification and tolerance.

This research exploits targeted metabolite assessment to elucidate As-responsive PC synthesis, exudation, and As–PC complexation in lupin. Chemical inhibition of GSH synthesis with BSO and ABC transporter-mediated exudation with vanadate are then used to elucidate complex exudation mechanisms and how they influence As fate in the rhizosphere.

## Materials and methods

### Plant growth and experimental design

**Fig. 1.**
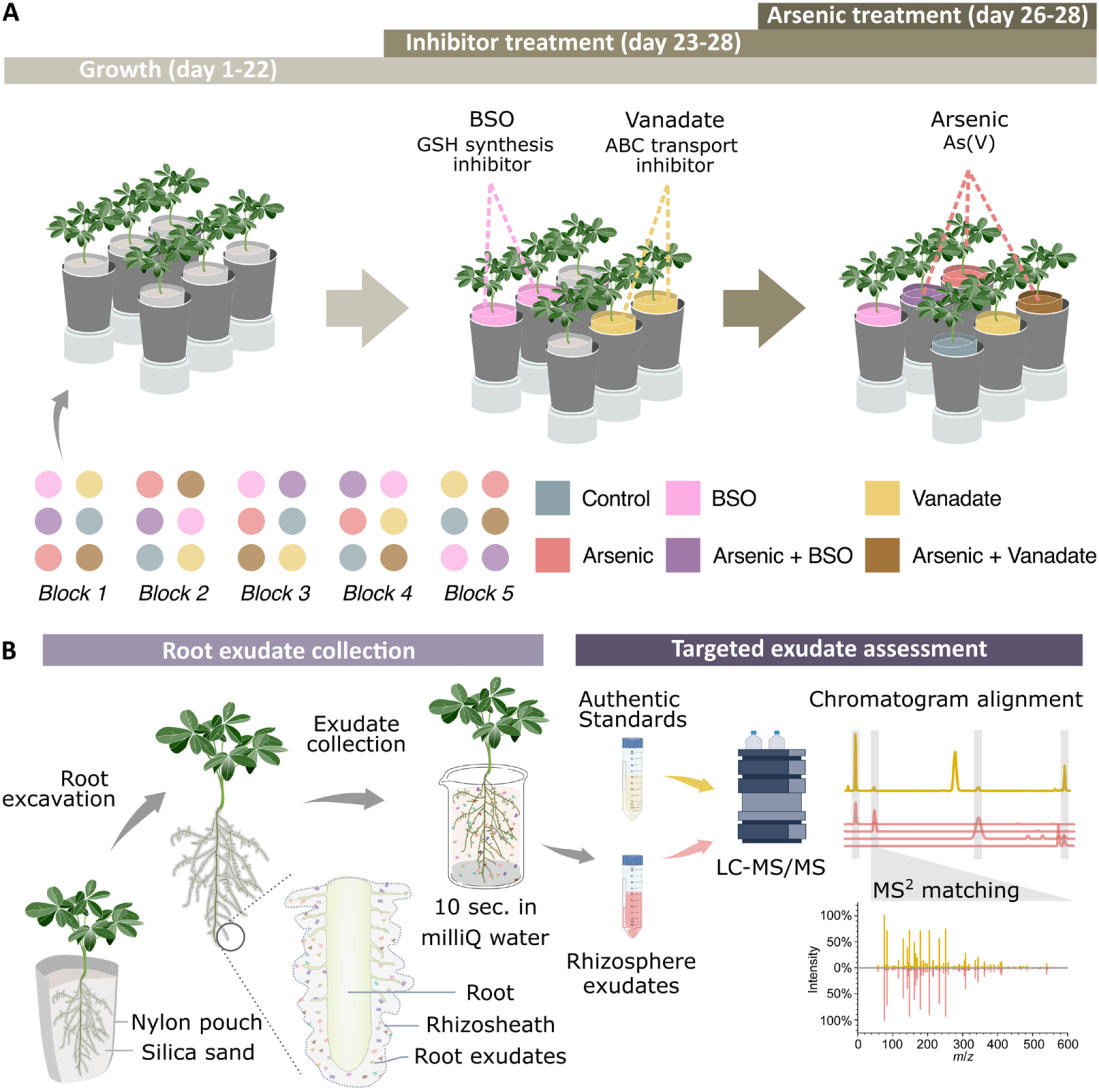
Experimental design and workflow for collecting and analysing root exudates. (A) Experimental design (*n*=5, illustrated; total plants=30), with each plant grown for 22 d before inhibitors, arsenic, and co-treatments. (B) Exudate capture workflow, adapted from Frémont *et al.* (2022), with permission from Wiley. Plants were extracted from nylon pouches with rhizosheaths intact, then briefly dipped in water to collect exudates. Exudate solutions were concentrated by freeze-drying before LC-MS/MS. Exudate metabolites were identified by comparing LC-MS/MS chromatograms and retention times with commercial standards and synthesized compounds (Supplementary Table S1), with definitive ID based on matching MS^2^ spectra to authentic standards.

Seeds of white lupin (*Lupinus albus* L. cv. AMIGA) were surface sterilized by sequential immersion in 70% ethanol, 1% sodium hypochlorite, and sterile Milli-Q water. Sterilized seeds were germinated on moist filter paper for 3 d, after which seedlings were transferred to growth pouches (7.6 × 15.2 cm) containing 700 ml of sterile silica sand, for a total of 30 plants divided into five blocks (Fig. 1A). Plants were supplied twice weekly with 65 ml of Hoagland nutrient solution (pH 6.0) containing the following concentrations: 600 μM KNO_3_, 400 μM Ca(NO_3_)_2_·4H_2_O, 200 μM NH_4_H_2_PO_4_, 100 μM MgSO_4_·7H_2_O, 5 μM KCl, 2.5 μM H_3_BO_3_, 0.2 μM MnSO_4_·H_2_O, 0.2 μM ZnSO_4_·7H_2_O, 0.05 μM CuSO_4_·5H_2_O, 0.05 μM H_2_MoO_4_, 4.5 μM Fe·Na·EDTA. Growth chamber conditions were 18 h photoperiod under LED lighting (150 μmol m^–2^ s^–1^) at 25 °C. At day 23 after planting, plants were either kept as untreated controls or subjected to BSO or vanadate inhibitor treatments (*n*=10 each) prepared in Hoagland solution: 0.5 mM BSO (>99% Thermo Fisher Scientific, Cat# AC235520010), a GSH synthesis inhibitor (Liu *et al.*, 2010), and 1 mM vanadate (sodium orthovanadate, >99% Thermo Fisher Scientific, Cat# AC205330500), an ABC-type transporter inhibitor (Song *et al.*, 2014). At day 26 after planting, plants were subjected to a second inhibitor treatment and further divided into + or – arsenate groups with the addition of 13 μM arsenate (Na_2_HAsO_4_·7H_2_O, sodium hydrogen arsenate heptahydrate, >98%, Thermo Fisher Scientific, Cat# AAA1827536), resulting in six treatments (*n*=5 each): control, arsenate, BSO, arsenate+BSO, vanadate, and arsenate+vanadate (Fig. 1A). Unplanted controls were also included for each treatment (*n*=5 each). Stomatal conductance was measured at day 27 and day 28 (24 h and 48 h after As addition, respectively) on the abaxial surface of the youngest fully expanded leaf (Vezza *et al.*, 2018) using a porometer (LI-600, LI-COR Biosciences). At harvest, 28 d after planting, shoot and root morphology, biomass, and root exudates were assessed.

### Root exudate collection and root metabolite extraction

Root exudates were collected following the procedure of Frémont *et al.* (2022). Briefly, nylon mesh growth pouches were opened and loose sand was gently removed, leaving intact root systems with attached rhizosheaths. Root systems were then immersed in 40 ml of Milli-Q water for 10 s with gentle circular agitation to collect exudates (Fig. 1B). Although some cellular injury may occur during sampling, this collection method was extensively validated to preclude artefacts from damage, as previously demonstrated (Frémont *et al.*, 2022). For unplanted controls, 20 g of sand from each unplanted pot was extracted under identical conditions to mirror the quantity of sand obtained from rhizosheaths. Solutions were lyophilized and stored at –70 °C. All lyophilized extracts were resuspended in 800 μl of Milli-Q water and 0.1% formic acid, filtered through 0.2 μm centrifuge filters (InnoSep Spin, Canadian Life Sciences) at 10 000 *g* for 30 s, transferred to HPLC vials, and kept at 4 °C before LC-MS/MS analysis within 24 h.

After rhizosphere extraction, whole-root systems went through five additional wash cycles in Milli-Q water with strong vortexing at maximum speed to recover root samples devoid of rhizosphere residues for endosphere metabolite extraction. Root samples were dried of residual water using absorbing paper, immediately frozen in liquid nitrogen, lyophilized, and kept at –70 °C. Root samples were ground using a mortar and pestle, and 50 mg of dried material was extracted in 1 ml of Milli-Q water+0.1% formic acid for 15 min with ultrasonication at 4 °C. Endosphere extracts were then centrifuged at 4 °C at 20 000 *g* for 2 min, the supernatant transferred to 0.2 μm centrifuge filters, centrifuged at 10 000 *g* for 30 s, transferred to HPLC vials, and kept at 4 °C before LC-MS/MS analysis within 24 h.

### Liquid chromatography–tandem mass spectrometry analysis

Targeted metabolite profiling was performed using an Agilent 1260 Infinity HPLC system coupled to an Agilent 6530 Q-TOF mass spectrometer with Jet Stream ionization source. Chromatographic separation utilized a Zorbax Eclipse Plus C18 column (4.6 × 100 mm, 3.5 μm) at 30 °C with a 0.4 ml min^–1^ flow rate. The 80 min gradient consisted of solvent A (5% methanol, 0.1% formic acid in water) and solvent B (methanol, 0.1% formic acid), starting at 100% A for 20 min, increasing linearly to 100% B over 50 min, then holding at 100% B for 10 min. Biological samples (*n*=30), unplanted controls (*n*=15), and Milli-Q water blanks (*n*=5) were analysed in ESI+ mode. MS^2^ fragmentation used a precursor ion inclusion list at 20 eV and 35 eV collision energy (Supplementary Table S1) and was performed on three samples per treatment (*n*=18). Source parameters included 300 °C gas temperature, 5 l min^–1^ drying gas, 310 kPa nebulizer pressure, 250 °C sheath gas temperature, and 11 l min^–1^ sheath gas flow.

LC-MS raw data were processed in MZmine 3.4.14 (Schmid *et al.*, 2023). Background noise was filtered at intensity thresholds of 800 for MS^1^ and 10 for MS^2^. Chromatograms were constructed within 10 ppm mass accuracy and minimum peak intensity of 800. Extracted ion chromatograms were deconvoluted using MZmine’s minimum search algorithm. Isotope peaks were grouped within 10 ppm mass tolerance and 0.1 min retention time tolerance. Features were aligned across samples within 15 ppm mass tolerance (75% weighting) and 0.2 min retention time tolerance (25% weighting). Features containing MS^2^ spectra were retained. Gaps in the feature matrix were filled within 0.8 min retention time and 5 ppm mass tolerances. MS^2^ spectra were matched to precursor ions within 0.02 *m/z* and 0.2 min retention time windows. MS^2^ spectra acquired at 20 V and 35 V collision energies were merged into consensus spectra after summing their intensities.

### Targeted metabolite annotation

Targeted features were annotated as GSH, glutathione disulfide (GSSG), PCs, and their As complexes based on matching retention times and MS^2^ spectra (cosine >0.8) to those of authentic standards analysed under identical LC-MS/MS conditions (Fig. 1B). The analytical work conducted here also does not discount compound transformation during sampling, such as the potential oxidation of GSH into GSSG, which could be collectively considered here as changes in total glutathione (but does not alter any conclusion presented). Standards were prepared from commercially obtained PC_2_ and PC_3_ (Anaspec, Fremont, CA, USA), GSH (Alfa Aesar Co., Inc., Ward Hill, MA, USA), and GSSG (ACROS Organics, Geel, Belgium). Oxidized PCs were generated via spontaneous oxidation during sample preparation. As–PC complexes were synthesized following Schmied-Tobies *et al.* (2014) by incubating PC_2_, PC_3_, and GSH standards (100 μM) with arsenite at various thiol (SH):As molar ratios (3:1, 1:3, and 1:6) in 0.1% formic acid. To synthesize the As–GS_3_ complex, GSH alone was incubated with As(III) at a 1:6 SH:As molar ratio. The full list of ions produced from As–PC *in vitro* complexation and their corresponding chemical formulas are shown in Supplementary Table S1.

### Statistical analysis

One-way ANOVA followed by Tukey’s honest significant difference (HSD) post-hoc test was used to determine treatment effects when data met assumptions of normality and homogeneity of variance. For non-normal or heteroscedastic data, the non-parametric Kruskal–Wallis test followed by Dunn’s post-hoc test was used for multiple comparisons. *P*-values were adjusted for false discovery rate control using the Benjamini–Hochberg method (Benjamini and Hochberg 1995). Pairwise comparisons were performed using *t*-tests when data met assumptions of normality and homogeneity of variance, and Mann–Whitney U tests for non-normal or heteroscedastic data. All analyses were conducted in R v.4.3.1 (R Core Team, 2020).

## Results

### Physiological response

**Fig. 2.**
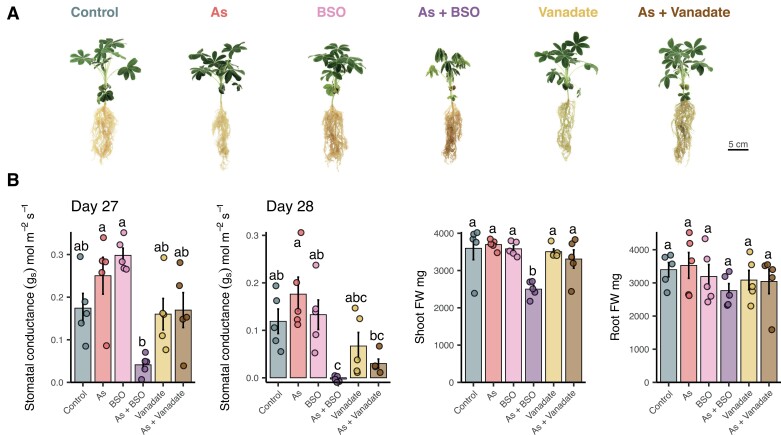
Physiological responses of white lupin to inhibitors, arsenic, and co-treatments. (A) Representative photographs of whole plants at harvest for each treatment. (B) Stomatal conductance (*g*_s_) 27 d and 28 d after planting (24 h and 48 h after As addition) and shoot and root fresh weight (FW) biomass at harvest. Data show means ±SE (*n*=5). Different letters indicate significant differences between treatments (ANOVA, Tukey HSD test, *P*<0.05).

Lupin plants (*L. albus*) either were left as untreated controls or were subjected to treatments with As, BSO, As+BSO, vanadate, or As+vanadate. Stomatal conductance (*g*_s_) was measured 27 d and 28 d after planting (24 h and 48 h after As addition, respectively). On day 27, neither As nor inhibitor treatments significantly affected *g*_s_, except for a significant decrease from 0.25 ± 0.043 mol m^−2^ s^−1^ to 0.04 ± 0.01 mol m^−2^ s^−1^ with As+BSO compared with As alone (*P*<0.05, ANOVA, Tukey’s HSD; Fig. 2B). By day 28, no significant effects on *g*_s_ were observed for As, BSO, or vanadate alone. However, As+BSO significantly reduced *g*_s_ to undetectable levels compared with 0.12 ± 0.026 mol m^−2^ s^−1^ in controls and 0.18 ± 0.036 mol m^−2^ s^−1^ in As-treated plants, and wilting was observed, while As+vanadate significantly decreased *g*_s_ to 0.03 ± 0.01 mol m^−2^ s^−1^ compared with As alone, without any observable morphological effects (Fig. 2A, B). At harvest on day 28, only As+BSO reduced shoot fresh biomass compared with controls, from 3596 ± 305 mg FW in controls to 2501 ± 98 mg FW in As+BSO (*P*<0.05, ANOVA, Tukey’s HSD).

### Targeted analysis of glutathione and phytochelatin exudates

**Fig. 3.**
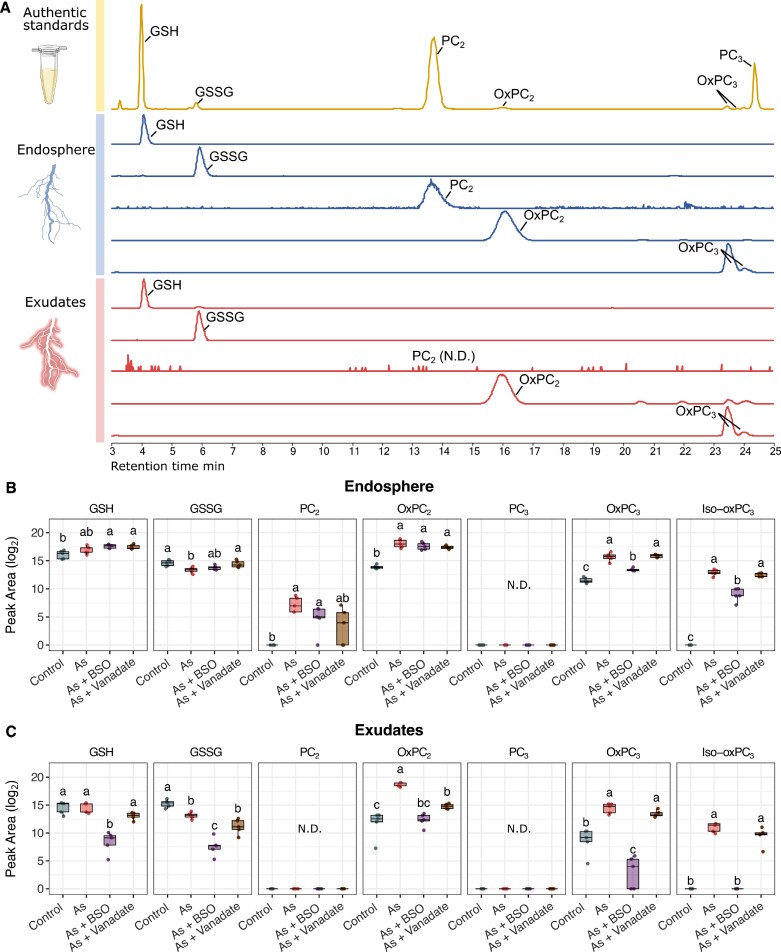
Glutathione derivatives and phytochelatins in the endosphere and root exudates in response to treatment. (A) Base peak chromatogram of glutathione (GSH) and phytochelatin (PC) standards (yellow) and extracted ion chromatograms of GSH (*m/z*: 308.09107; [M+H]^+^), oxidized glutathione (GSSG) (*m/z*: 613.15862; [M+H]^+^), PC_2_ (*m/z*: 540.14152; [M+H]^+^), oxidized PC_2_ (oxPC_2_) (*m/z*: 538.12685; [M+H]^+^), oxPC_3_, and its isomer (iso-oxPC_3_) (*m/z*: 770.17546; [M+H]^+^) detected in the endosphere (blue) and exudates (red) of As-treated lupin plants, with definitive ID based on matching retention time and MS^2^ spectra. (B) GSH derivatives and PC abundance (log2 peak area) in the endosphere (root extracts) from controls and treated plants. (C) GSH derivatives and PC abundance in exudates from controls and treated plants. For all boxplots, the bottom and top of the boxes correspond to the lower and upper quartiles, and the centre line marks the median (*n*=5). Different letters indicate significant differences between treatments (ANOVA, Tukey HSD test, *P*<0.05). Full factorial plots including all six treatments are presented in Supplementary Fig. S1. MS^1^ and MS^2^ spectra for each compound are presented in Supplementary Fig. S2. Abbreviations: As, arsenic; BSO, l-buthionine sulfoximine; N.D., not detected.

To evaluate the metabolic response of lupin to As, root tissue (endosphere) extracts and rhizosphere exudates from As-treated plants were analysed using LC-MS/MS and compared with authentic standards. Six major glutathione-derived compounds and PCs were measured: GSH, GSSG, PC_2_, oxidized PC_2_ (oxPC_2_), PC_3_, and two isomers of oxidized PC_3_ (oxPC_3_ and iso-oxPC_3_). The endosphere contained GSH, GSSG, both reduced and oxidized forms of PC_2_, but only oxPC_3_ (Fig. 3A). Exudates contained GSH, GSSG, oxPC_2_, and oxPC_3_. OxPC_2_ was most prominent in both the endosphere and rhizosphere of As-treated plants.

In comparing the endosphere of control and As-treated plants, GSH levels were not significantly different, while GSSG was depleted in As-treated roots (Fig. 3B). PC_2_ was detected in As-treated plants but not in controls, while PC_3_ was not detected. OxPC_2_ and oxPC_3_ were enriched in As-treated roots compared with controls, with iso-oxPC_3_ not detected in controls (Fig. 3B). In exudates, GSH did not significantly vary between controls and As-treated plants, while GSSG was significantly depleted (Fig. 3C). Exuded oxPC_2_ and oxPC_3_ were enriched in As-treated plants compared with controls, with oxPC_2_ the most enriched compound, and iso-oxPC_3_ absent in untreated controls (Fig. 3C).

### Inhibition of arsenic response

In the endosphere, co-treatment with As+BSO did not significantly alter levels of GSH, GSSG, PC_2_, and oxPC_2_ compared with As alone (Fig. 3B). However, oxPC_3_ and iso-oxPC_3_ were significantly depleted with As+BSO co-treatment compared with As alone. Compared with controls, GSH, PC_2_, oxPC_2_, oxPC_3_, and iso-oxPC_3_ were significantly enriched in As+BSO co-treatment, while GSSG did not significantly vary. Co-treatment with As+vanadate resulted in no significant change in levels of GSH, PC_2_, oxPC_2_, oxPC_3_, and iso-oxPC_3_ compared with As alone, but induced a significant enrichment of GSSG. Compared with control, As+vanadate significantly increased GSH, oxPC_2_, oxPC_3_, and iso-oxPC_3_, but did not significantly affect levels of GSSG or PC_2_.

In exudates, co-treatment with As+BSO significantly reduced the As response of the thiol-containing compounds, with GSH, GSSG, and oxPC_3_ being depleted compared with both control and As treatment, while oxPC_2_ and iso-oxPC_3_ returned to control levels (Fig. 3C). Co-treatment with As+vanadate did not significantly alter GSH, GSSG, oxPC_3_, and iso-oxPC_3_ levels compared with As treatment, but did significantly deplete levels of oxPC_2_. Compared with controls, As+vanadate co-treatment resulted in similar levels of GSH and a significantly depleted level of GSSG, as well as significantly increased levels of oxPC_2_, oxPC_3_, and iso-oxPC_3_ (Fig. 3C).

### Targeted analysis of arsenic–phytochelatin complexes in exudates

**Fig. 4.**
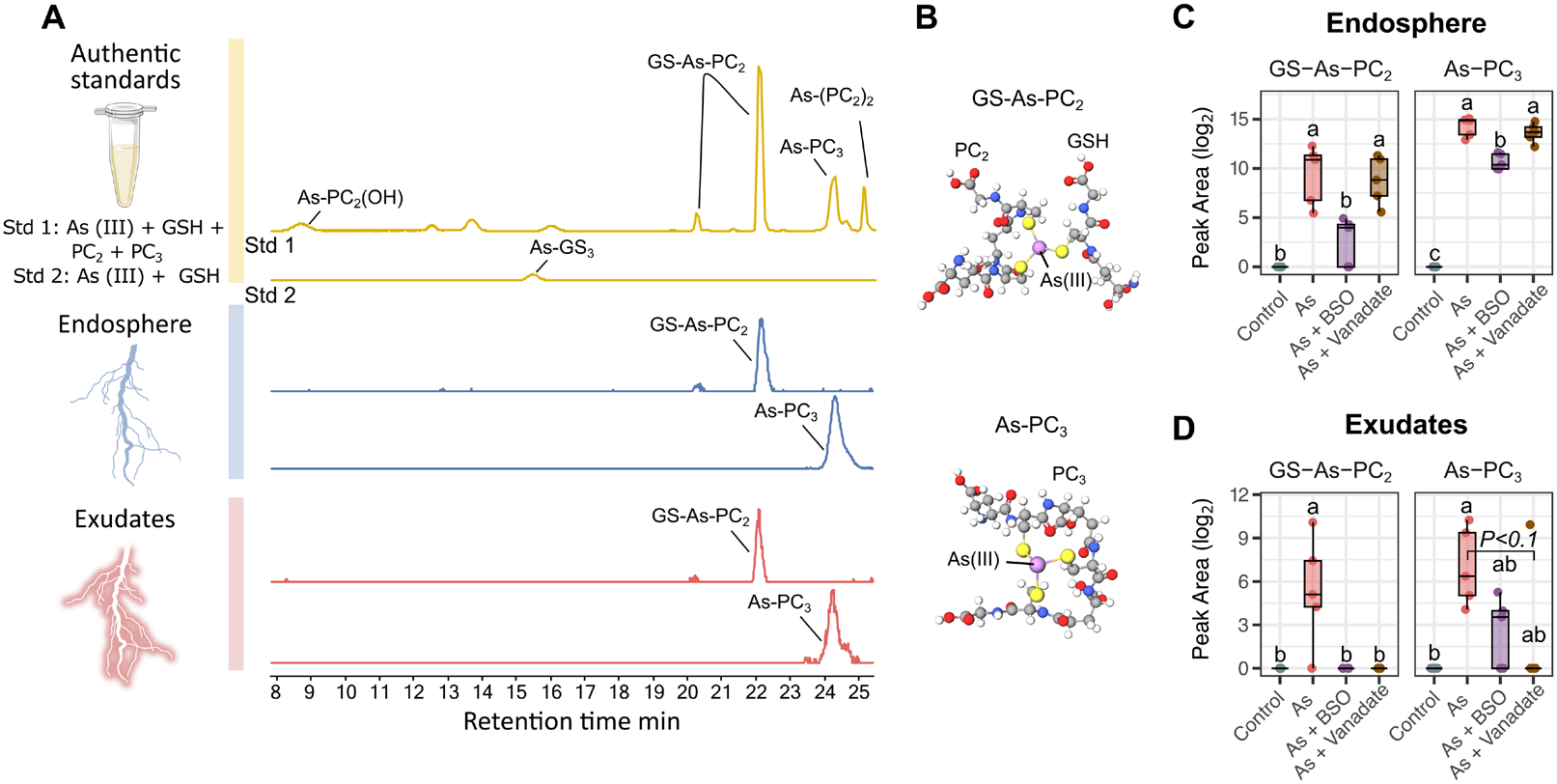
Arsenic–phytochelatin complexes in the endosphere and root exudates in response to treatments. (A) Base peak chromatogram of synthesized As–PC standards (yellow) and extracted ion chromatograms of GS–As–PC_2_ (*m/z*: 460.06593; [M+2H]^2+^) and As–PC_3_ (*m/z*: 844.09144; [M+H]^+^) detected in the endosphere (blue) and exudates (red) of As-treated lupin plants, with definitive ID based on matching retention time and MS^2^ spectra (Supplementary Fig. S2). (B) Proposed structures of As–PCs in As-treated lupin, adapted from Schmied-Tobies *et al.* (2014), with permission from Wiley. 3D models generated using MolView (https://molview.org/). (C) Abundance (log2 peak area) of As–PCs in root endosphere from control and treated plants. (D) As–PC abundance in exudates from control and treated plants. For all boxplots, the bottom and top of the boxes correspond to the lower and upper quartiles, and the centre line marks the median (*n*=5). Different letters indicate significant differences between treatments (ANOVA, Tukey HSD test; Kruskal–Wallis, Dunn’s test; *P*.adj <0.05). Full factorial plots including all six treatments are presented in Supplementary Fig. S3. MS^1^ and MS^2^ spectra for each compound are presented in Supplementary Fig. S4. Abbreviations: As, arsenic; BSO, l-buthionine sulfoximine.

To evaluate the potential for detecting As–PC complexes in the endosphere and root exudates, an *in vitro* experiment was conducted in which arsenite As(III) was incubated in 0.1 M formic acid with standards of GSH, PC_2_, and PC_3_, either individually or in combinations at varying thiol:As molar ratios. LC-MS/MS revealed six chromatographic peaks present only with As addition, corresponding to five distinct As–PC complexes and their isomers (Fig. 4A; Supplementary Table S1).

By re-analysing endosphere and exudate samples with these *in vitro* derived spectra (Fig. 4A), two As–PC complexes were identified in both the endosphere and exudates: GS–As–PC_2_, which comprised one molecule of GSH and one molecule of PC_2_ coordinated to As(III) through their three thiol groups, and As–PC_3_, in which As(III) is coordinated to the three thiol groups of one PC_3_ molecule (Fig. 4B). GS–As–PC_2_ and As–PC_3_ were absent from control endosphere and exudate samples, and As–PC_3_ was the most prevalent As complex in the As-treated endosphere and exudates (Fig. 4C, D).

In the endosphere, addition of BSO significantly reduced levels of both GS–As–PC_2_ and As–PC_3_ compared with As treatment alone, while vanadate had no significant effect on endosphere GS–As–PC_2_ and As–PC_3_ levels compared with As treatment alone (Fig. 4C). However, both the application of BSO and the application of vanadate eliminated all GS–As–PC_2_ from exudates, while As–PC_3_ exudation was only partially reduced with BSO, and almost eliminated with vanadate, with only one outlier with detectable As–PC_3_ (Fig. 4C, D).

## Discussion

### Combination of metabolic inhibitors and arsenic disrupts lupin arsenic tolerance

Arsenic alone had no significant effect on stomatal conductance or biomass in lupin, which confirms tolerance to short-term exposure to 13 μM As(V) as previously observed (Vázquez *et al.*, 2005; Frémont *et al.*, 2022). Similarly, the application of inhibitors without As treatment did not affect physiological parameters compared with control, except for a small non-significant increase in stomatal conductance 24 h after BSO treatment, indicating that physiological functions were largely maintained during inhibitor treatment. However, co-treatment with As and BSO, which inhibits GSH synthesis (Liu *et al.*, 2010), substantially reduced both stomatal conductance and biomass, particularly after 48 h, with complete loss of stomatal conductance and wilting (Fig. 2). This acute sensitivity to As+BSO co-treatment resembles the response previously reported with much higher (5×) As concentrations (Frémont *et al.*, 2022), underscoring the protective role of GSH and derivatives in mitigating As toxicity in lupin. Additionally, co-treatment with As and vanadate, an ATP-dependent membrane transport inhibitor (Song *et al.*, 2014), decreased stomatal conductance at 48 h, highlighting vanadate-induced As sensitivity and suggesting the involvement of active transmembrane transport in lupin As detoxification.

### Targeted exudate assessment reveals critical roles of glutathione-derived metabolites and phytochelatin synthesis and exudation in lupin arsenic tolerance

In a previous study, Frémont *et al.* (2022) reported the presence of PCs in exudates of As-treated lupin plants. Since PCs are major As detoxification metabolites in plants (Cobbett and Goldsbrough, 2002), the exudation of PCs and their GSH precursor was examined here to gain insight into their mechanisms of exudation, interactions with As, and roles in As detoxification. With the exception of GSH, which was unchanged, all measured GSH derivatives and PCs in the endosphere and exudates exhibited significant and substantial responses to As addition compared with controls (Fig. 3). GSSG decreased by 8% in the endosphere and by 17% in As-treated exudates. Since GSH is challenging to capture in exudates owing to its high reactivity (Giustarini *et al.*, 2016), the significant GSSG depletion is an important clue indicating a general shift of the GSH pathway towards increased PC synthesis in response to As (Vázquez *et al.*, 2005; Frémont *et al.*, 2022). Oxidized PCs (oxPC_2_ and oxPC_3_) increased in abundance by >30% in the endosphere and >60% in exudates, confirming the enriched synthesis and exudation of oxPC_2_ in response to As reported by Frémont *et al.* (2022) and the novel detection of oxPC_3_ in lupin exudates.

Surprisingly, while As+BSO co-treatment significantly decreased GSH exudation, it did not decrease GSH levels in the endosphere (Fig. 3B), contradicting the target role of BSO as an endogenous GSH synthesis inhibitor (Liu *et al.*, 2010). An explanation for this could be that absolute production levels of GSH were reduced but concentrations were inflated against the resulting reduced growth. Another possible explanation could be the reduction of GSH exudation, compensating for the BSO-mediated reduction in endogenous GSH synthesis in order to maintain GSH levels within roots. Different responses between the endosphere and exudates were also observed for GSSG and oxPC_2_, which remained unchanged in the endosphere but were drastically reduced in exudates upon addition of As+BSO co-treatment (Fig. 3C), suggesting a reduced GSH availability affecting exudation of metabolites downstream from GSH, such as GSSG, and PCs. On the other hand, the significant depletion of oxPC_3_ in the endosphere and in exudates with As+BSO co-treatment indicates decreased PC_3_ synthesis and exudation. Owing to the fact that PC_3_ is a product of the stepwise condensation of γ-Glu-Cys moieties to PC_2_ itself and of the growing PC chain, its synthesis is dependent on the availability of both PC_2_ and GSH (Grill *et al.*, 1989), which is likely to be strongly compromised with the addition of As+BSO. The severe disruption of lupin As tolerance with As+BSO observed here (Fig. 2) suggests that inhibiting GSH synthesis would be likely to have disrupted PC production and exudation, which compromised essential As detoxification mechanisms such as the chelation of As(III) into non-toxic As–PC complexes.

Inhibition of ATP-dependent membrane transport with vanadate effectively reduced exudate levels of oxPC_2_ by more than 22% but did not influence endogenous synthesis (Fig. 3). This indicates that active transport and exudation of oxPC_2_ across membranes would be likely to involve (ATP-dependent) ABC transporters, analogous to those involved in As–PC complex loading into vacuoles in *A. thaliana* (Song *et al.*, 2010). However, unaltered levels of oxPC_3_ in the endosphere and exudates after As+vanadate treatment suggests that a different exudation route may exist for these compounds. Collectively, although plasma membrane ABC transport appears important to facilitate oxPC_2_ exudation, this alone may not fully explain the observed As sensitivity with As–vanadate co-treatment (Fig. 2) but is likely to represent one of multiple As detoxification mechanisms conferring lupin As tolerance, which potentially include As–PC complex exudation.

### Exudation of phytochelatin–arsenic complexes provides a new route for arsenic efflux and detoxification in lupin

Characterizing As–PC complexes in plant matrices presents several analytical challenges (Bluemlein *et al.*, 2008, 2009) and is even more difficult in complex extracellular environments such as the rhizosphere. To target specific As–PCs in the endosphere and the rhizosphere, As–PC complexes were first synthesized *in vitro* from GSH, PC_2_, and PC_3_, and analysed using LC-MS/MS (Fig. 4A). Five distinct As–PC species were detected, representing all known As–PC coordination schemes from these three compounds (Bluemlein *et al.*, 2009; Schmied‐Tobies *et al.*, 2014). A targeted search for these complexes in the endosphere and exudates of As-treated plants revealed the presence of two As–PC complexes (Fig. 4A, B), indicating that rhizosphere As complexation, or As complex exudation from roots, may act as an as yet unknown As detoxification mechanism in lupin.

**Fig. 5.**
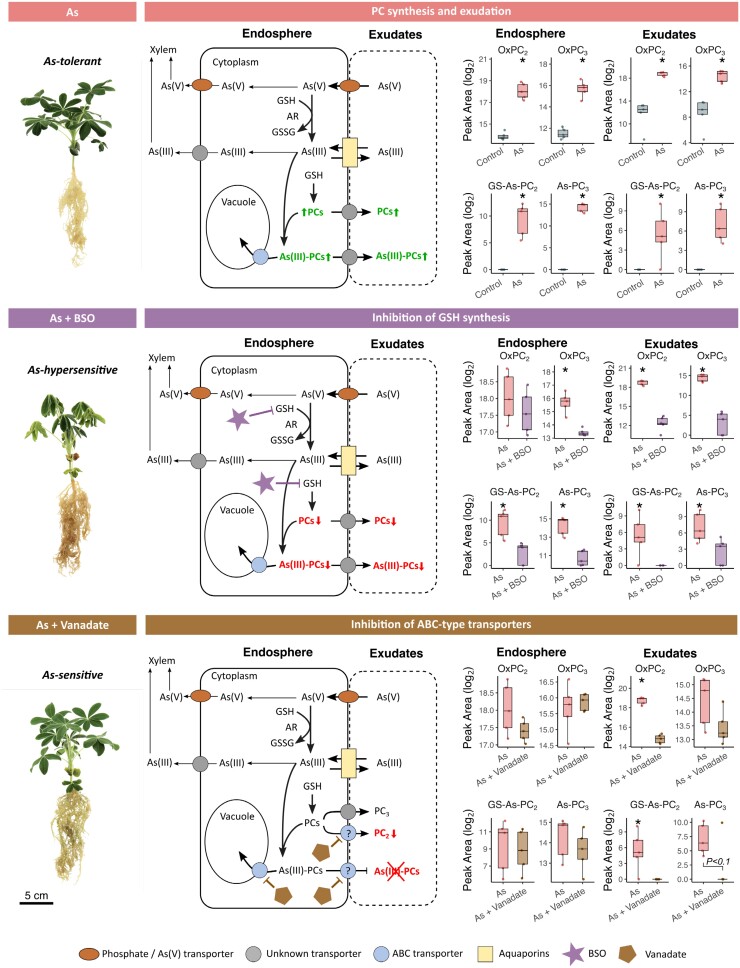
Arsenic-treated *Lupinus albus* plants after chemical inhibition and corresponding putative detoxification models. Adapted from Li *et al.* (2016), by permission of Oxford University Press. Arrows indicate demonstrated or putative As metabolic and transport pathways, while question marks highlight knowledge gaps. For all boxplots, the bottom and top of the boxes correspond to the lower and upper quartiles, and the centre line marks the median (*n*=5); asterisks denote significant differences (*P*<0.05; *t*-test or Mann–Whitney U test). The three plant images are enlarged duplications from Fig. 2A, provided as context to data here. Abbreviations: As, arsenic; BSO, l-buthionine sulfoximine; ABC, ATP-binding cassette transporter; AR, arsenate reductase; GSSG, glutathione disulfide; GSH, glutathione; PC, phytochelatin.

Using this As–PC complex fingerprint, complexation in the rhizosphere was also explored after the use of detoxification inhibitors. By disrupting GSH synthesis, BSO also interrupted or drastically reduced As–PC in the endosphere and in exudates (Fig. 4C, D), indicating that As detoxification through As–PC complex formation relies on the availability of GSH for PC synthesis and As binding (Grill *et al.*, 1989). Conversely, vanadate treatment had no effect on endosphere As–PC levels but abolished As–PC complexes in exudates (Fig. 4C, D); this is likely to be due to inactivation of target membrane ABC-type transporters. This provides evidence of As–PC exudation as an active process, potentially occurring via ATP-dependent ABC transporters, similar to those plants used for As–PC vacuolar loading and sequestration (Song *et al.*, 2010) (Fig. 5). In *A. thaliana* and *Oryza sativa*, As–PC transport from the cytosol to the vacuole is mediated by an ABCC-type transporter system (Song *et al.*, 2010, 2014). These transporters may also mediate As–PC exudation to the rhizosphere, as suggested by the immunofluorescence localization of ABCC transporters to outer root cell layers in Song *et al.* (2014). This new route for As detoxification in lupin may provide an additional mechanism beyond the current understanding of As(III) efflux from roots, of which ~20% is explained by NIP transporter-mediated efflux of free As(III), while the remaining 80% is still unaccounted for (Zhao *et al.*, 2010b). While there is some evidence of increased unbound As(III) efflux when PC production is compromised (Liu *et al.*, 2010), to our knowledge, this is the first investigation and report of As–PC complexes in exudates and characterization of the exudation mechanisms involved.

### Limitations and future work

Accurate sampling and assessment of root exudate compounds can be challenging due to the complexity of rhizosphere environments, the wide diversity and low concentrations of metabolites, and technical limitations in detection and quantification methods (Escolà Casas and Matamoros, 2021; Salem *et al.*, 2022), particularly for potentially transient exuded As–PC complexes. Contemporary metabolite assessment approaches rely on direct comparison of spectra for accurate detection of metabolite changes between treatments in complex sample matrices, but provide limited insight into the relative contribution of a metabolite to a specific putative functional pathway. Estimated absolute concentrations of PCs and As–PC (Supplementary Table S2) provide some further insight into the potential As detoxification contribution. Determining the proportional contribution of an As–PC exudation pathway to As detoxification and As speciation dynamics in the rhizosphere is an exciting research priority raised by these findings, in order to reveal the potential to influence metal tolerance. Similarly, future studies should explore if this pathway is conserved in other crops, model organisms, and with other metals.

These findings indicate that As–PC complexes may contribute to As(III) efflux from roots, providing a new As detoxification and tolerance mechanism in plants. In addition to potentially contributing to the 80% of previously unexplained As(III) exudation from roots (Zhao *et al.*, 2010b), this pathway may have different specificity for As (and other toxic metals such as cadmium) compared with aquaporin channels. Ultimately, a better understanding of this new pathway and its contribution to overall As detoxification could help optimization of phytoremediation applications and inform strategies aimed at reducing As accumulation in food crops.

## Supplementary data

The following supplementary data are available at *JXB* online.

Table S1. Glutathione, phytochelatin, and arsenic–phytochelatin species identified from standards and *in vitro* complexation assay using LC-MS/MS.

Table S2. Glutathione, phytochelatin, and arsenic–phytochelatin species identified in endosphere and exudate samples using LC-MS/MS.

Fig. S1. GSH derivatives and PCs in the endosphere and root exudates in response to treatment.

Fig. S2. MS^1^ and MS^2^ spectra of GSH derivatives and PCs in the endosphere and root exudates.

Fig. S3. As–PC complexes in the root endosphere and exudates in response to treatments.

Fig. S4. MS^1^ and MS^2^ spectra of As–PC complexes in the endosphere and root exudates.

erae272_suppl_Supplementary_Materials

## Data Availability

All primary data to support the findings of this study were deposited in MassIVE and are openly available at https://massive.ucsd.edu/ProteoSAFe/static/massive.jsp; MSV000093078. Spectra from As–PC *in vitro* complexation were added as new library entries on the Global Natural Products Social Molecular Networking (GNPS) platform (https://gnps.ucsd.edu), and spectra IDs are listed in Supplementary Table S1.

## Abbreviations

ABC: ATP-binding cassette transporter
AR: arsenate reductase
BSO: L-buthionine sulfoximine
GSH: glutathione
GSSG: glutathione disulfide
HSD: honest significant difference
oxPC: oxidised phytochelatin
PC: phytochelatin
