# Supplementary material for: Arsenic stress triggers active exudation of arsenic–phytochelatin complexes from Lupinus albus roots
Source: J Exp Bot. 2024 Jun 12;75(18):5897–908. doi: 10.1093/jxb/erae272 (PMC11427844; doi:10.1093/jxb/erae272)
Supplement: erae272_suppl_Supplementary_Materials [file erae272_suppl_supplementary_materials.pdf]

Table S1 Glutathione, phytochelatin, and arsenic-phytochelatin species identified from standards and in vitro complexation assay using liquid chromatography-tandem mass spectrometry (LC-MS/MS). This list was used for targeted analysis of endosphere and exudate samples via LC-MS/MS. Global Natural Products Social Molecular Networking (GNPS) spectrum IDs correspond to new entries in the GNPS open spectral libraries (accessible at https://library.gnps2.org/).

Precursor m/z	Adduct	Mean RT (min)	Compound name	Molecular formula	GNPS Spectrum ID
**308.092**	$[M+H]^+$	3.5	GSH	C10H17N3O6S	-
**613.160**	$[M+H]^+$	5.6	GSSG	C20H32N6O12S2	-
**630.049**	$[M+H]^+$	8.6	As-PC_2(OH)	C18H28AsN5O11S2	CCMSLIB00010011905
**540.143**	$[M+H]^+$	13.4	PC_2	C18H29N5O10S2	CCMSLIB00010011902
**497.583**	$[M+2H]^{2+}$	15.0	$As(GS)_3$	C30H48AsN9O18S3	CCMSLIB00010011946
**994.157**	$[M+H]^+$	15.0	$As(GS)_3$	C30H48AsN9O18S3	CCMSLIB00010011947
**538.127**	$[M+H]^+$	15.7	oxPC_2	C18H27N5O10S2	CCMSLIB00010011944
**460.067**	$[M+2H]^{2+}$	20.1	GS-As-PC_2	C28H43AsN8O16S3	-
**919.125**	$[M+H]^+$	20.1	GS-As-PC_2	C28H43AsN8O16S3	-
**919.125**	$[M+H]^+$	22.0	GS-As-PC_2	C28H43AsN8O16S3	CCMSLIB00010173329
**460.067**	$[M+2H]^{2+}$	22.0	GS-As-PC_2	C28H43AsN8O16S3	CCMSLIB00010173330
**844.092**	$[M+H]^+$	24.1	As-PC_3	C26H38AsN7O14S3	CCMSLIB00010011906
**422.550**	$[M+2H]^{2+}$	24.2	As-PC_3	C26H38AsN7O14S3	CCMSLIB00010011906
**770.178**	$[M+H]^+$	23.3	oxPC_3	C26H39N7O14S3	CCMSLIB00010011945
**772.192**	$[M+H]^+$	24.3	PC_3	C26H41N7O14S3	CCMSLIB00010127002
**576.092**	[M+2H]2+	25.2	$As(PC_2)_2$	C36H55AsN10O20S4	CCMSLIB00010011949
**1151.175**	$[M+H]^+$	25.4	$As(PC_2)_2$	C36H55AsN10O20S4	CCMSLIB00010011948

Table S2 Glutathione, phytochelatin, and arsenic-phytochelatin species identified in endosphere and exudate samples using liquid chromatography-tandem mass spectrometry (LC-MS/MS). Putative concentrations were calculated from As-treated samples as PC2 equivalents and should be interpreted cautiously.

Precursor m/z	Adduct	Mean RT (min)	Compound name	Molecular formula	Theoretical m/z	Putative concentrations in exudates (ng g root DW in PC_2 equivalents)
**308.091**	$[M+H]^+$	3.9	GSH	C10H17N3O6S	308.091	22.3 ± 2.62
**613.158**	$[M+H]^+$	5.8	GSSG	C20H32N6O12S2	613.159	7.87 ± 1.13
**538.127**	$[M+H]^+$	16.1	oxPC_2	C18H27N5O10S2	538.127	375 ± 28.8
**770.175**	$[M+H]^+$	23.6	oxPC_3	C26H39N7O14S3	770.179	19.2 ± 2.77
**770.177**	$[M+H]^+$	25.3	oxPC_3	C26H39N7O14S3	770.179	1.79 ± 0.21
**460.066**	$[M+2H]^{2+}$	22.1	GS-As-PC_2	C28H43AsN8O16S3	460.066	0.17 ± 0.12
**844.091**	[M+H]+	24.3	As-PC_3	C26H38AsN7O14S3	844.093	0.25 ± 0.13

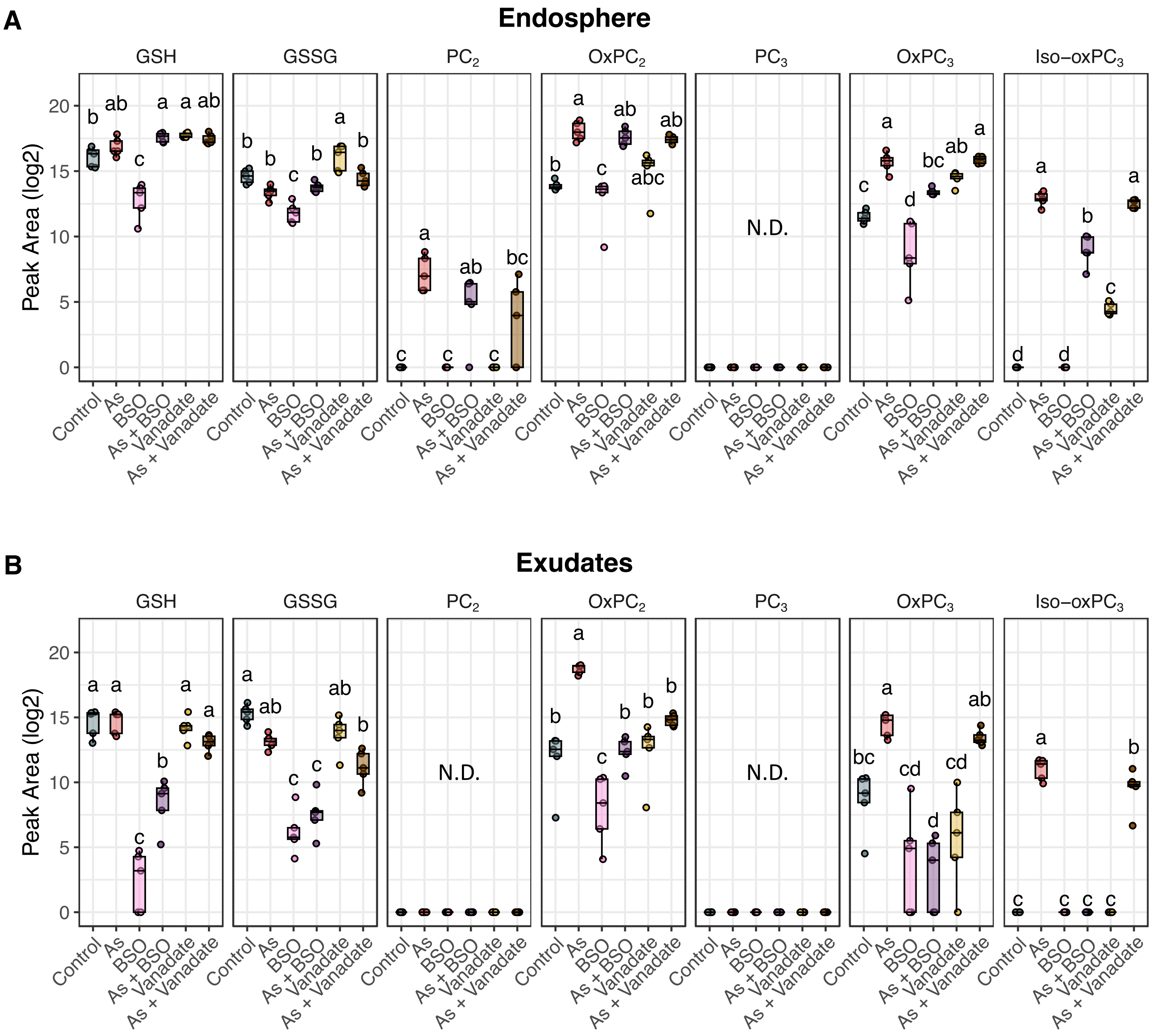

Fig. S1 GSH derivatives and PCs in the root endosphere and exudates in response to treatment. **a** PC and GSH derivatives abundance (Log2 peak area) in endosphere (root extracts) from controls and treated plants. **b** PC and GSH derivatives abundance in exudates from controls and treated plants. For all boxplots the bottom and top of the boxes correspond to the lower and upper quartiles and the center line marks the median (n = 5). Different letters indicate significant differences between treatments (ANOVA, Tukey HSD test, $P < 0.05$).

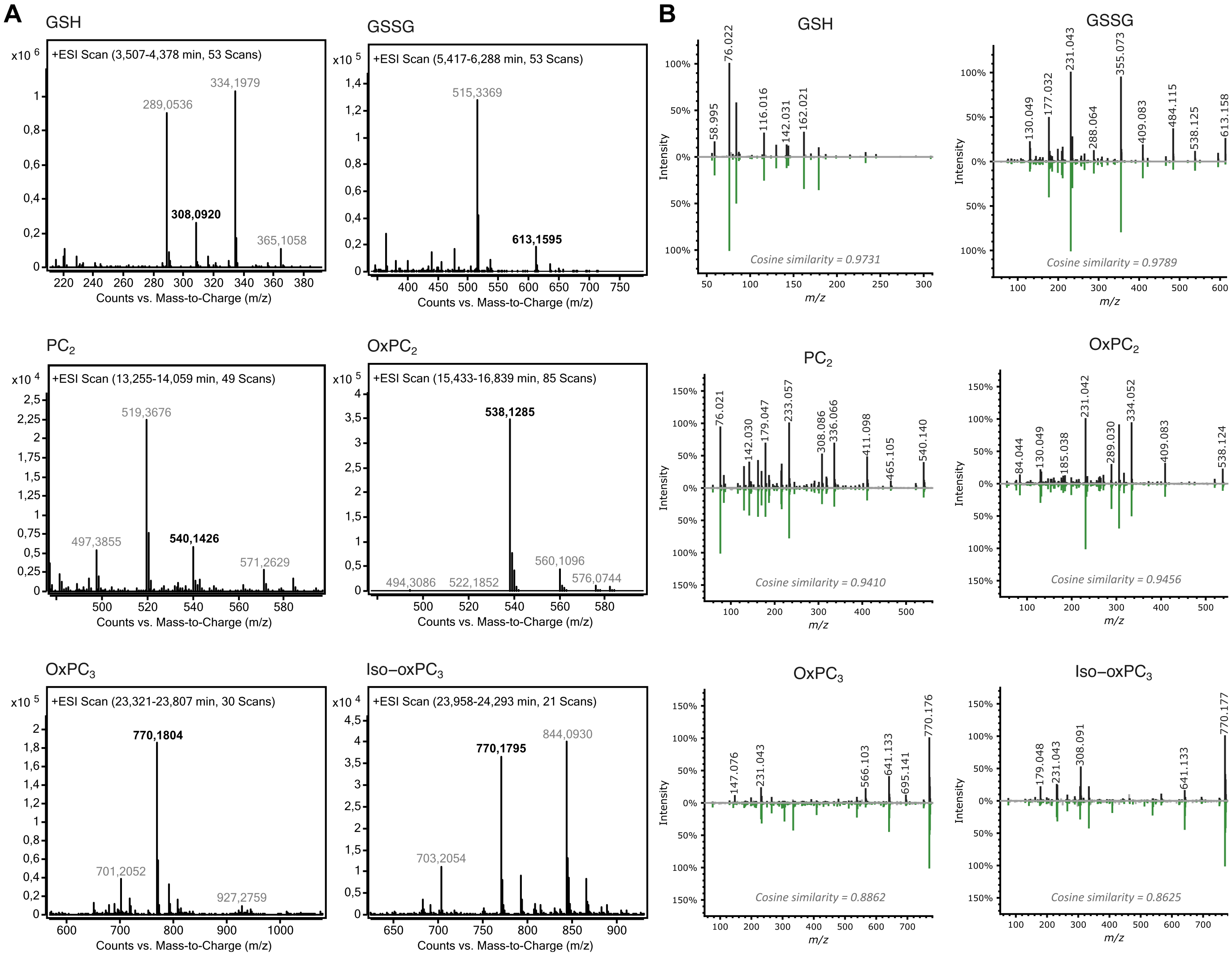

Fig. S2 MS[1] and MS[2] spectra of GSH derivatives and PCs in the root endosphere and exudates. (A) MS[1] spectra of PC and GSH derivatives. **(B)** Mirror plots of MS[2] spectra of PC and GSH derivatives from samples (black), compared to standards (green). Matching tandem mass spectrometry (MS/MS) spectra with cosine scores >0.8 were used to confirm metabolite identities.

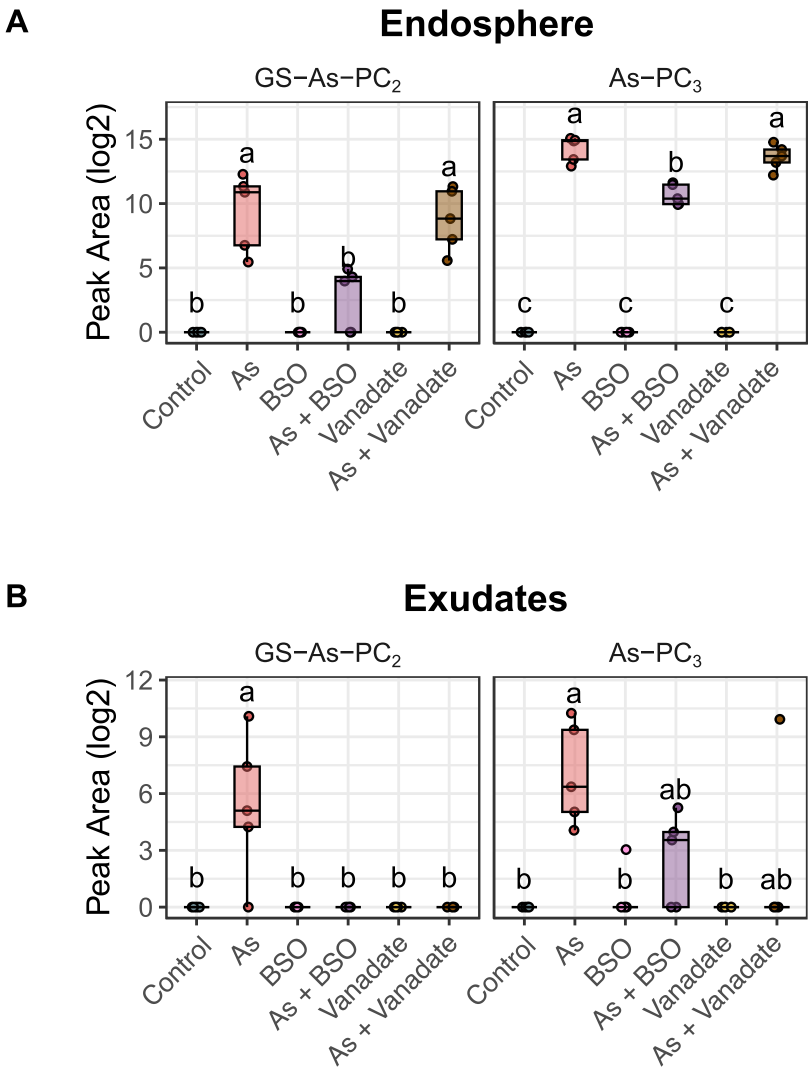

Fig. S3 Arsenic-phytochelatin (As-PC) complexes in the root endosphere and exudates in response to treatments. a Mirror plots comparing As-PCs synthesized in vitro to those detected in exudates. Matching tandem mass spectrometry (MS/MS) spectra with cosine scores >0.9 were used to confirm metabolite identities. **b** Abundance (Log2 peak area) of As-PCs in root endosphere from control and treated plants. **b** As-PC abundance in exudates from control and treated plants. For all boxplots the bottom and top of the boxes correspond to the lower and upper quartiles and the center line marks the median (n = 5). Different letters indicate significant differences between treatments (ANOVA, Tukey HSD test; Kruskal-Wallis, Dunn's test; p.adj < 0.05).

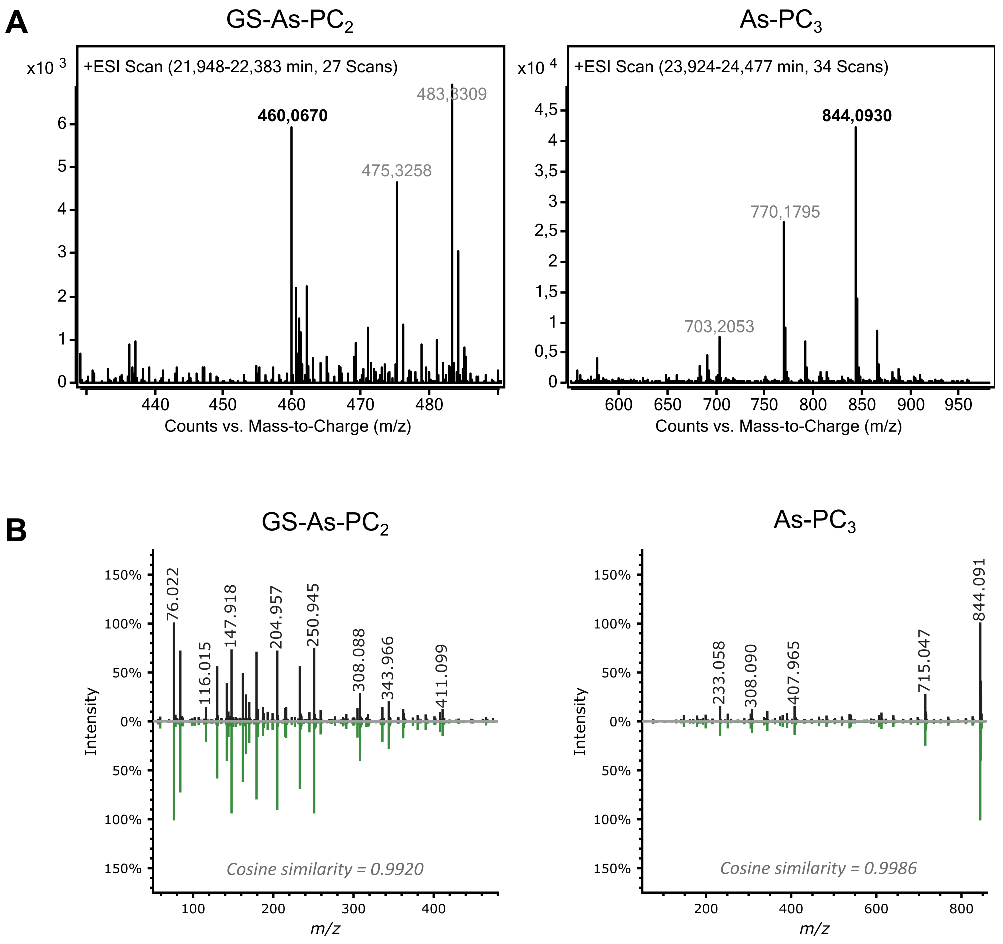

Fig. S4 MS[1] and MS[2] spectra of As-PC complexes in endosphere and root exudates. (A) MS[1] spectra of As-PC complexes. **(B)** Mirror plots of MS[2] spectra As-PC complexes from samples (black), compared to standards (green). Matching tandem mass spectrometry (MS/MS) spectra with cosine scores >0.8 were used to confirm metabolite identities.
